# The role of SRPK1-mediated phosphorylation of SR proteins in the chromatin configuration transition of mouse germinal vesicle oocytes

**DOI:** 10.7555/JBR.38.20240054

**Published:** 2024-05-29

**Authors:** Xia Wang, Shuai Zhou, Haojie Yin, Jian Han, Yue Hu, Siqi Wang, Congjing Wang, Jie Huang, Junqiang Zhang, Xiufeng Ling, Ran Huo

**Affiliations:** 1 State Key Laboratory of Reproductive Medicine and Offspring Health, Department of Histology and Embryology, Suzhou Affiliated Hospital of Nanjing Medical University, Suzhou Municipal Hospital, Gusu School, Nanjing Medical University, Nanjing, Jiangsu 211166, China; 2 Department of Reproductive Medicine, Women's Hospital of Nanjing Medical University, Nanjing Women and Children's Healthcare Hospital, Nanjing, Jiangsu 210004, China

**Keywords:** oocyte, chromatin, nuclear speckle, SR protein, phosphorylation, SRPK1

## Abstract

Meiotic resumption in mammalian oocytes involves nuclear and organelle structural changes, notably the chromatin configuration transition from a non-surrounding nucleolus (NSN) to surrounding nucleolus (SN) in germinal vesicle oocytes. In the current study, we found that nuclear speckles (NSs), a subnuclear structure mainly composed of serine-arginine (SR) proteins, changed from a diffuse spotted distribution in mouse NSN oocytes to an aggregated pattern in SN oocytes. We also found that the SR protein-specific kinase 1 (SRPK1), an enzyme that phosphorylates SR proteins, co-localized with NSs at the SN stage, and that NSN oocytes failed to transition to SN oocytes after the inhibition of SRPK1 activity. Furthermore, the typical structure of the chromatin ring around the nucleolus in SN oocytes collapsed after treatment with an SRPK1 inhibitor. Mechanistically, phosphorylated SR proteins were found to be related to chromatin as shown by a salt extraction experiment, and *in situ* DNase I assay showed that the accessibility of chromatin was enhanced in SN oocytes when SRPK1 was inhibited, accompanied by a decreased repressive modification on histone and the abnormal recurrence of a transcriptional signal. In conclusion, our results indicated that SRPK1-regulated phosphorylation of SR proteins was involved in the NSN-SN transition and played an important role in maintaining the condensed nucleus of SN oocytes *via* interacting with chromatin.

## Introduction

The morphological and physiological changes in the nucleus are essential for mammalian oocytes during the transition from the germinal vesicle (GV) stage to the metaphase Ⅱ (MⅡ) stage^[1–2]^. Based on their heterogeneous chromatin configuration, oocytes are categorized into two subpopulations: the non-surrounding nucleolus (NSN) and the surrounding nucleolus (SN)^[3]^. These two classes of oocytes have uneven subsequent developmental potential, with fewer NSN oocytes reaching the MⅡ stage and more frequently arresting at the preimplantation stage, compared with SN oocytes^[4]^. Furthermore, transcription is active in NSN oocytes, whereas it is silenced at the SN stage, as assessed by BrUTP incorporation^[5]^. Some transcriptome results reflect the differences between NSN and SN oocytes, indicating that SN oocytes are more competent for embryogenesis^[6–7]^. Additionally, nuclear staining reveals significant differences in the morphology of the nucleus between NSN and SN oocytes, which in turn affects the distribution of macromolecules and structures in the nucleus^[8]^. For instance, fibrillarin displays a heterogeneous and cloudy localization in the nucleus of NSN oocytes, but it exists an even distribution as bright dots in SN oocytes^[9]^. In addition, the distribution of acetylated histones shifts from a scattered pattern to the nucleolar surface region as NSN oocytes transition to SN oocytes^[10]^. However, the differences in the physiological state between these two stages and the underlying molecular mechanisms remain unclear.

Nuclear speckles (NSs), which are a kind of membraneless body located in the interchromatin region, play an important role in transcription, pre-mRNA splicing, and nuclear export of mRNAs^[11–12]^. The high dynamics of NSs are observed in mammalian somatic cells, with dissolution during the mitotic phase and reformation following late telophase^[13]^. However, the distribution of NSs in germ cells is still unclear. Furthermore, the components of NSs are constantly in flux^[14]^. Although it has been reported that NSs contain two essential components, SRRM2 and SON, and that the deletion of these in somatic cells leads to the disappearance of the NS structure^[15]^, there is no consensus on the composition of NS. Studies have shown that NSs are mainly composed of a class of proteins containing serine-arginine repeat domain (*i.e.*, SR proteins)^[16]^. Therefore, an antibody targeting SC35 (also known as SRSF2) is typically used to identify the NS structure.

Many forms of post-transcriptional modification have been identified on SR proteins, including methylation, acetylation, and phosphorylation^[17]^. The phosphorylation of SR proteins is related to almost all biological activities in which SR proteins are involved^[18–19]^. Several kinases catalyze the phosphorylation of serine residues in serine-arginine dipeptide motifs within SR proteins^[17]^, including SR protein-specific kinase 1 (SRPK1). It is reported that SRPK1 initiates the protamine-to-histone exchange by catalyzing site-specific phosphorylation of protamine during embryogenesis^[20]^, but its role during oocyte maturation remains unclear.

In the current study, we investigated whether the SR protein-enriched NSs were altered during the meiotic process, especially between the NSN and SN stages, and examined the involvement of SRPK1 in regulating the phosphorylation of SR proteins.

## Materials and methods

### Oocyte collection and culture

GV oocytes were collected from unstimulated four- to six-week-old ICR female mice in accordance with the approval from the Institutional Animal Care and Use Committee of Nanjing Medical University, Jiangsu, China (IACUC-2006046). Ovaries were harvested and punctured in M2 medium (Sigma, St. Louis, MO, USA) containing 2.5 μmol/L milrinone (Sigma), and oocytes surrounded by cumulus cells were exclusively collected and denuded mechanically.

GV oocytes were cultured in M2 medium containing 2.5 μmol/L milrinone for 15 h at 37 ℃ under 5% CO_2_ *in vitro* to induce meiotic arrest. Then, they were washed in milrinone-free M16 medium (Sigma) at least three times and cultured in M16 medium for 4 h to assess the ratio of germinal vesicle breakdown (GVBD) and for 14 h to assess the ratio of the first polar body (Pb1) extrusion. To distinguish the chromatin organization of NSN and SN, freshly denuded oocytes were stained with Hoechst 33258 (Sigma) at 37 ℃ for 5 min and sorted under a fluorescent stereoscope (Nikon, Tokyo, JPN). SRPKIN-1 (MedChemExpress, Monmouth Junction, NJ, USA), a covalent and irreversible SRPK1 inhibitor, was used to block the SRPK1-mediated phosphorylation of SR proteins for 15 h during the period of meiotic arrest, with an equal volume of dimethyl sulfoxide (DMSO, Sigma) added as a negative control.

### Isolation of nuclei and chromatin fractionation

Cytoplasmic and nuclear fractions were isolated using the nuclear and cytoplasmic extraction kit (BestBio, Beijing, China) according to the manufacturer's instructions. Then, the nuclei were suspended in chromatin elution buffer (10 mmol/L Tris [pH 7.4], 2 mmol/L MgCl_2_, 2 mmol/L EGTA, and 0.1% Triton X-100), and increasing concentrations of NaCl (70 mmol/L) were used for sequential incubation at 4 ℃ for 30 min. The nuclei were centrifuged at 400 *g* for 30 min to release the weakly chromatin-bound proteins into the supernatant. To collect protein fractions with different chromatin-binding strengths, the pellet was resuspended in chromatin elution buffer containing NaCl at concentrations of 70, 150, 300, and 600 mmol/L. Western blotting was performed to detect the binding properties of the target protein to chromatin.

### Western blotting

Fifty GV oocytes with a defined chromatin configuration or treated with SRPKIN-1 for 15 h were harvested and lysed in the RIPA lysis buffer (CWBio, Taizhou, Jiangsu, China) containing the protease inhibitor and phosphatase inhibitor cocktail (CWBio), and then incubated on ice for 30 min with a combination of 20% sodium dodecyl sulfate (SDS) sample buffer (FDBIO, Hangzhou, Zhejiang, China). The lysate was boiled for 5 min, separated by 10% SDS-PAGE (FDBIO), and transferred onto PVDF membranes (Millipore, Billerica, MA, USA). The membranes were blocked in tris buffered saline (TBS) with 0.1% Tween-20 (TBST) and 5% skim milk at room temperature for 2 h before incubating with primary antibody at 4 ℃ overnight. The primary antibodies used in the current study were as follows: anti-SRPK1 (1∶200; Cat. #6111072, BD Biosciences, Franklin, NJ, USA), anti-phospho-SR proteins (1∶500; Cat. #MABE50, Sigma), anti-β-actin (1∶10000; Cat. #AC026, ABclonal, Wuhan, Hubei, China), and anti-GAPDH (1∶5000; Cat. #AC002, ABclonal). After three washes with TBST, the membranes were incubated with corresponding secondary antibody, HRP goat anti-rabbit IgG (H+L) (1∶5000; Cat. #31460, Invitrogen, Carlsbad, CA, USA) or HRP goat anti-mouse IgG (H+L) (1∶5000; Cat. #31430, Invitrogen), at room temperature for 2 h. The protein bands were visualized using an enhanced chemiluminescence kit (FDBIO) and detected using the Bio-Rad gel imaging system.

### Immunofluorescence staining

Oocytes, either freshly denuded or treated with SRPKIN-1 for 15 h, were fixed in 4% paraformaldehyde (PFA, Sigma) for 2 h and then permeabilized in phosphate-buffered saline (PBS) with 0.5% Triton X-100 for 30 min. After blocking in the buffer containing 5% bovine serum albumin for 1 h, oocytes were immunostained with primary antibodies at 4 ℃ overnight. Antibodies used were as follows: anti-SC35 (1∶200; Cat. #S4045, Sigma), anti-SRPK1 (1∶200; Cat. #ab131160, Abcam, Cambridge, UK), anti-histone H3 lysine 9 trimethylation (H3K9me3; 1∶200; Cat. #ab8898, Abcam), anti-histone H3 lysine 27 trimethylation (H3K27me3; 1∶200; Cat. #9733, CST, Danvers, MA, USA), anti-α-tubulin (1∶500; Cat. #322588, Invitrogen), and anti-γH2AX (1∶500; Cat. #ab22551, Abcam). Next, the samples were washed three times in PBS and labeled with secondary antibody, Alexa Fluor 555 donkey anti-rabbit IgG (H+L) (1∶500; Cat. #A-31572, Invitrogen) or Alexa Fluor 488 donkey anti-mouse IgG (H+L) (1∶500; Cat. #A-21202, Invitrogen), at room temperature for 1 h, followed by Hoechst 33258 staining for 5 min, and briefly washed in PBS. Finally, oocytes were transferred to the anti-fade medium (VectorLabs, Newark, CA, USA) on glass slides and observed using an LSM710 laser confocal microscope (ZEISS, Jena, Germany). The detection of NS was measured by the maximum intensity projections of SC35 immunostaining, where 8 z-stacks were acquired for each image with a step size of 6 μm. All other fluorescence results were obtained by photographing the equatorial section of each oocyte.

The signal intensity of γH2AX, H3K9me3, and H3K27me3 was quantified by taking the mean intensity of the whole nucleus as readout by the ZEN software (blue edition, ZEISS). The fluorescence signals of SC35, SRPK1, and α-tubulin were calculated through the location, volume, or form by the ImageJ software.

### DNase I-TUNEL assay

GV oocytes treated with SRPKIN-1 for 15 h were collected and washed twice in PBS, permeabilized in extraction buffer (50 mmol/L NaCl, 3 mmol/L MgCl_2_, 0.5% Triton X-100, and 300 mmol/L sucrose in 25 mmol/L HEPES, pH 7.4) on ice for 5 min, washed twice in extraction buffer without Triton X-100, and then transferred to extraction buffer with 1 U/mL DNase I (Roche, Basel, Switzerland) at 37 ℃ for 5 min. Finally, the oocytes were fixed with 2% PFA at room temperature for 10 min. The TUNEL assay was performed using the DeadEnd Fluorometric TUNEL System (Roche), following the manufacturer's protocols. All samples were stained with Hoechst 33258 for 5 min. The TUNEL signal was detected by confocal microscopy, and the quantification of mean intensity in each oocyte nucleus was performed using the ZEN software.

### TUNEL assay

After the SRPKIN-1 treatment, GV oocytes were collected and washed twice in PBS, and then permeabilized in extraction buffer (50 mmol/L NaCl, 3 mmol/L MgCl_2_, 0.5% Triton X-100, and 300 mmol/L sucrose in 25 mmol/L HEPES, pH 7.4) on ice for 5 min. Subsequently, the samples were washed twice in extraction buffer without Triton X-100. The DeadEnd Fluorometric TUNEL System was used according to the manufacturer's instructions. All samples were stained with Hoechst 33258 for 5 min. TUNEL signal was observed and quantified as described above.

### EU assays

The SRPKIN-1-treated GV oocytes were cultured in SRPKIN-1-free M16 medium with 1 mmol/L EU (Invitrogen) for 2 h and fixed in 4% PFA in PBS at room temperature for 30 min. The incorporation of EU into the new synthetic RNA was detected using the Click-iT RNA Alexa Fluor 594 Imaging Kit (Invitrogen) according to the manufacturer's instructions. The number of EU foci was detected using confocal laser scanning microscopy.

### Statistical analysis

Data were presented as mean ± standard error of the mean. Statistical significance was determined by the unpaired two-tailed Student's *t*-test using GraphPad Prism 7.0 software (GraphPad, San Diego, CA, USA). *P* < 0.05 was considered significant.

## Results

### The distribution of NSs dynamically changed in mouse oocytes during meiotic maturation

**Figure 1:**
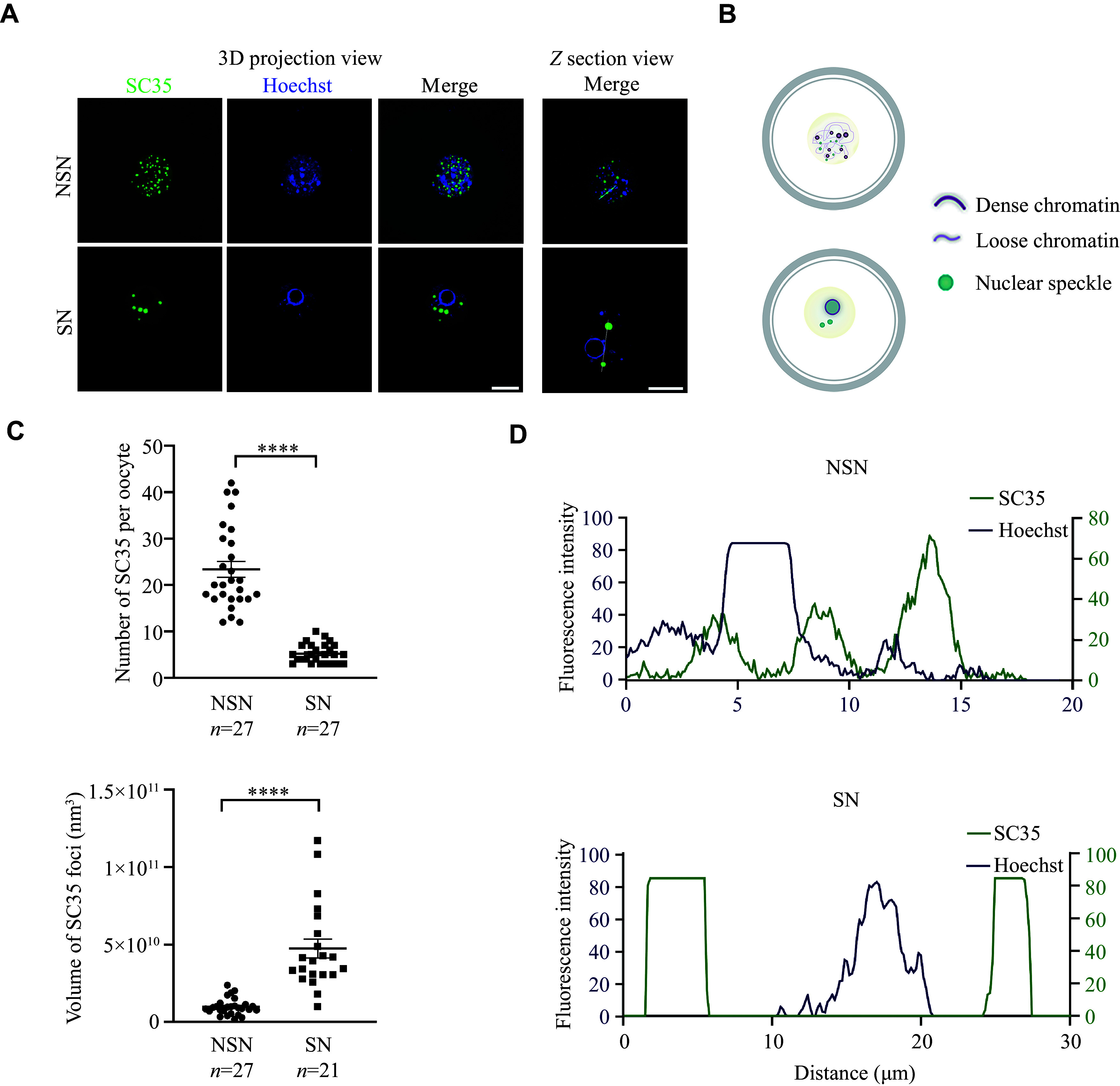
The distribution of NSs in mouse oocytes during meiotic maturation. A: Representative 3D projection and z-section confocal images of SC35 and Hoechst 33258 in denuded freshly NSN and SN oocytes. Scale bar, 20 µm. B: A schematic illustration of chromatin condensation and NS distribution change during the NSN to SN transition. C: Quantification of the number and volume of the SC35 signal in 3D projection confocal images of NSN and SN oocytes from panel A. D: Analysis of the colocalization of Hoechst 33258 and SC35 signals in NSN and SN oocytes from panel A. Data are presented as mean ± standard error of the mean. ^****^P < 0.0001 by the unpaired two-tailed Student's *t*-test. Abbreviations: NSs, nuclear speckles; NSN, non-surrounding nucleolus; SN, surrounding nucleolus.

Since the regulatory mechanism of NSs in germ cells is poorly studied, we investigated the characteristics of NS distribution and function during mouse oocyte development. To visualize NSs in GV oocytes, we performed immunofluorescence staining against SC35, a well-established marker of NS, also known as SRSF2^[21]^. The signal of SC35 displayed an intranuclear dispersive punctate pattern in NSN oocytes, but was distributed around the chromatin ring in SN oocytes, showing much more clustered and rounded structures (***Fig. 1A*** and ***1B***). After the maximum projection of all stacks, NSs were found to be larger but fewer in number in SN oocytes than in NSN oocytes (***Fig. 1C***). We then analyzed the colocalization of Hoechst 33258 and SC35, and observed that SC35-positive loci were related to low chromatin density, but were distributed closely to the chromatin in NSN oocytes (***Fig. 1A*** and ***1D***). Collectively, these results demonstrate the high variation of NSs in mouse oocytes and indicate their potential involvement in chromatin condensation during meiosis resumption.

### SR proteins were phosphorylated by SRPK1 in oocytes

**Figure 2:**
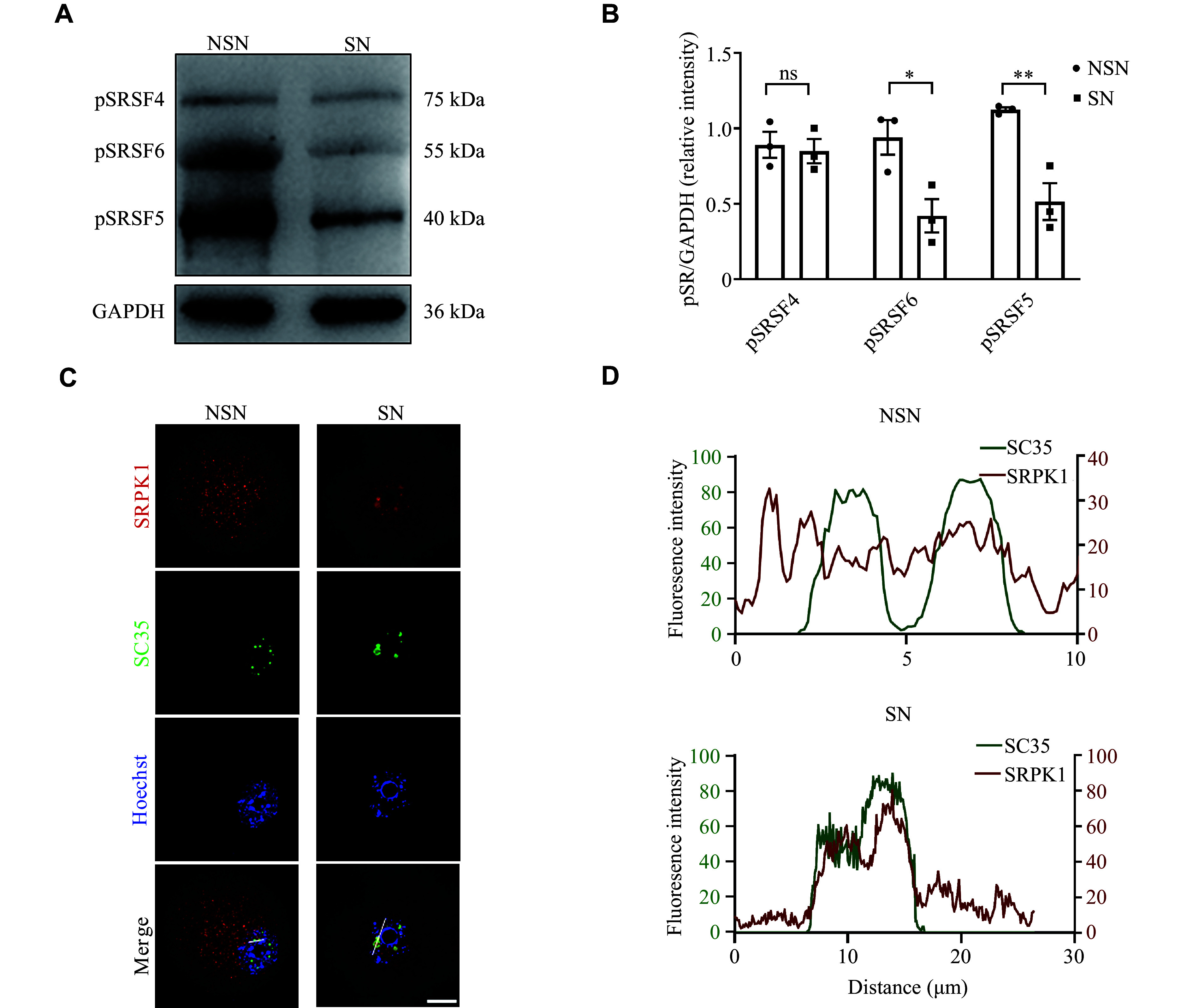
Serine-arginine (SR) proteins were phosphorylated by SRPK1 in oocytes. A: Western blotting showing the levels of pSR (probed as pSRSF) in GV oocytes with defined chromatin configuration. GAPDH was used as the loading control. B: Quantification of the grayscale from the panel A (*n* = 3 independent replicates). C: Representative staining images showing the distributions of SRPK1 and SC35 in NSN and SN oocytes. Scale bar, 20 µm. D: Comparison of colocalization between SC35 and SRPK1 signal in the nucleus at NSN and SN oocytes. Data are presented as mean ± standard error of the mean. ^*^P < 0.05 and ^**^P < 0.01 in unpaired two-tailed Student's *t*-test. Abbreviations: NSN, non-surrounding nucleolus; SN, surrounding nucleolus; SRPK1, SR protein-specific kinase 1; pSR, phosphorylated SR; GAPDH, glyceraldehyde-3-phosphate dehydrogenase; ns, not significant.

SR proteins usually aggregate in NS and play a regulatory role through phosphorylation. Therefore, we detected the phosphorylation levels of SR proteins. Western blotting analysis showed that the levels of phosphorylated SR proteins (pSR) decreased from the NSN stage to the SN stage, especially in pSRSF5 and pSRSF6 **(*****Fig. 2A*** and ***2B***). Additionally, we detected the SRPK1 in GV oocytes because of its high expression and its role in catalyzing the phosphorylation of SR proteins. Although SRPK1 exhibited comparable expression levels between NSN and SN oocytes (***Supplementary Fig. 1A*** and ***1B***, available online), we observed a diffuse distribution of SRPK1 in the cytoplasm and nucleus at the NSN stage, but mainly concentrated in the nucleus at the SN stage (***Fig. 2C***). Furthermore, the nuclear localization of SRPK1 was noticed to colocalize with NS only in SN oocytes but not in NSN oocytes (***Fig. 2D***). We then used SRPKIN-1, an SRPK1 inhibitor that forms a specific covalent bond with the tyrosine phenol group in the kinase's ATP-binding pocket^[22]^, to determine whether SRPK1 mediates the phosphorylation of SR proteins in GV oocytes. A significant dose-dependent reduction in pSR levels was observed, when GV oocytes were cultured with 20 and 50 μmol/L SRPKIN-1 for 15 h (***Supplementary Fig. 1C*** and ***1D***). Accordingly, 50 μmol/L was selected as the optimum concentration for subsequent experiments. When NSN oocytes were cultured in the medium supplemented with SRPKIN-1, NSs were more clustered (***Supplementary Fig. 1E*** and ***1F***). However, the size of NS in SN oocytes treated with SRPKIN-1 did not change significantly (***Supplementary Fig. 1G*** and ***1H***). These results demonstrate that dynamically distributed SRPK1 kinase may regulate the phosphorylation of SR proteins, thereby affecting the state of NS in GV oocytes.

### Inhibition of the phosphorylation of SR proteins impeded oocyte maturation *in vitro*

**Figure 3:**
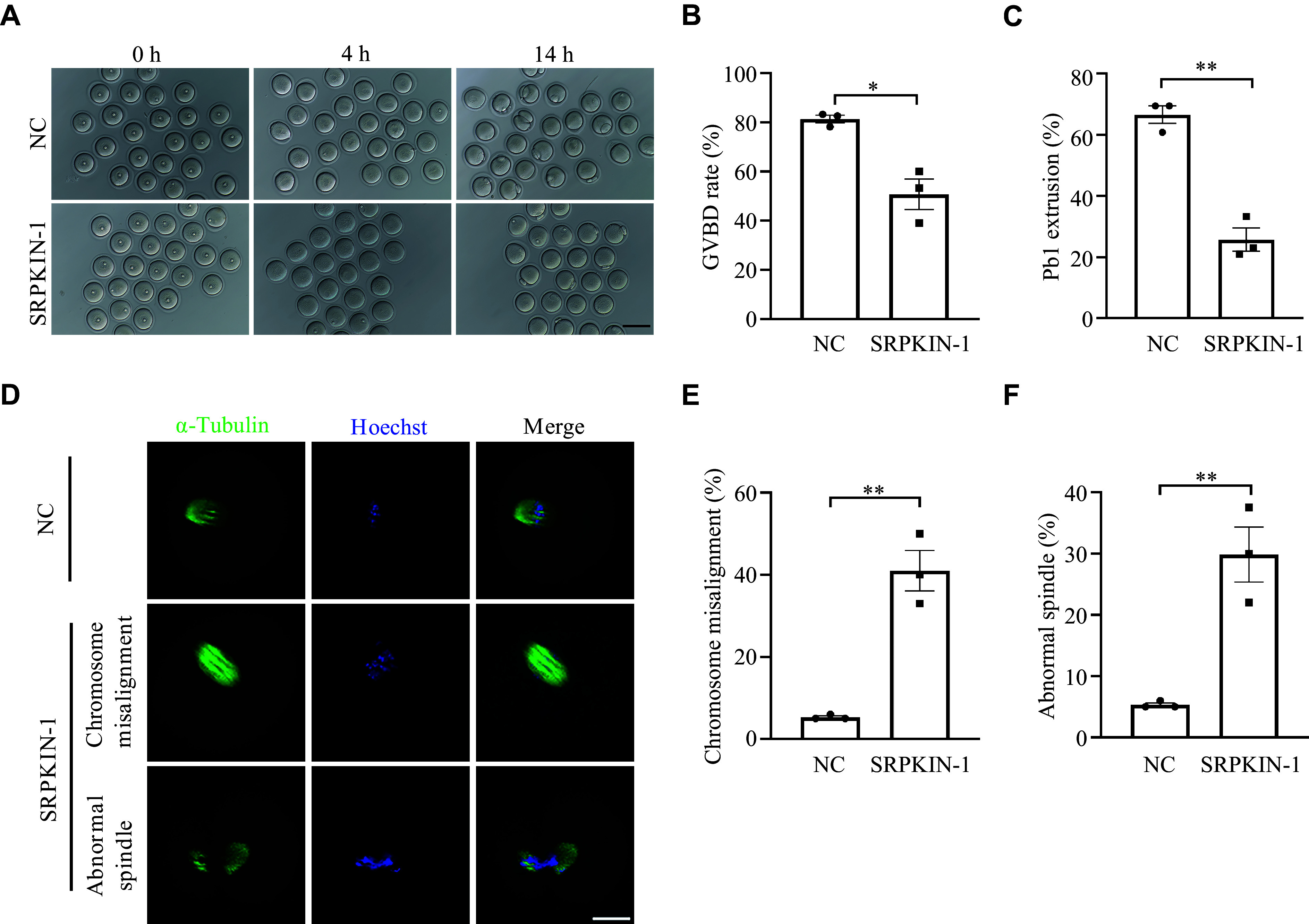
SRPKIN-1 inhibited oocyte maturation *in vitro*. A: Representative differential interference contrast images of GV oocytes cultured in M16 medium for 4 h and 14 h with SRPKIN-1 (50 μmol/L) or an equal volume of dimethyl sulfoxide (NC). Scale bar, 100 µm. B and C: Quantitative analysis of GVBD (B) and Pb1 extrusion rate (C) in NC and SRPKIN-1 treated oocytes. A total of 72 and 83 oocytes were included in the NC and SRPKIN-1 treatment groups, respectively (*n* = 3 independent replicates). D: Representative images of spindle and chromosome of MⅡ oocytes developed *in vitro* after SRPKIN-1 treatment. Scale bar, 20 μm. E and F: Percentage of oocytes with chromosome misalignment (E) and abnormal spindles (F) in the two groups. A total of 75 and 63 oocytes were included in the NC and SRPKIN-1 treatment groups, respectively (*n* = 3 independent replicates). Data are presented as mean and standard error of the mean. ^*^P < 0.05 and ^**^P < 0.01 by the unpaired two-tailed Student's *t*-test. Abbreviations: SRPKIN-1, SR protein-specific kinase irreversible inhibitor 1; GV, germinal vesicle; GVBD, GV breakdown; Pb1, the first polar body; NC, negative control; MⅡ, metaphase Ⅱ.

To investigate the influence of SRPK1-mediated phosphorylation of SR proteins on oocyte maturation, we treated GV oocytes with SRPKIN-1 to observe GVBD and examine the Pb1 extrusion (***Fig. 3A***). The results showed that SRPKIN-1 treatment led to a significant decrease in the percentage of GVBD and Pb1 extrusion (***Fig. 3B*** and ***3C***), but a significant increase in the frequencies of abnormal spindles, accompanied by misaligned chromosomes in oocytes (***Fig. 3D***–***3F***), compared with the control group. These results suggest that the meiotic maturation may be adversely influenced when the phosphorylation of SR proteins is suppressed.

### Phosphorylation of SR proteins was associated with chromatin in NSN-SN transition

**Figure 4:**
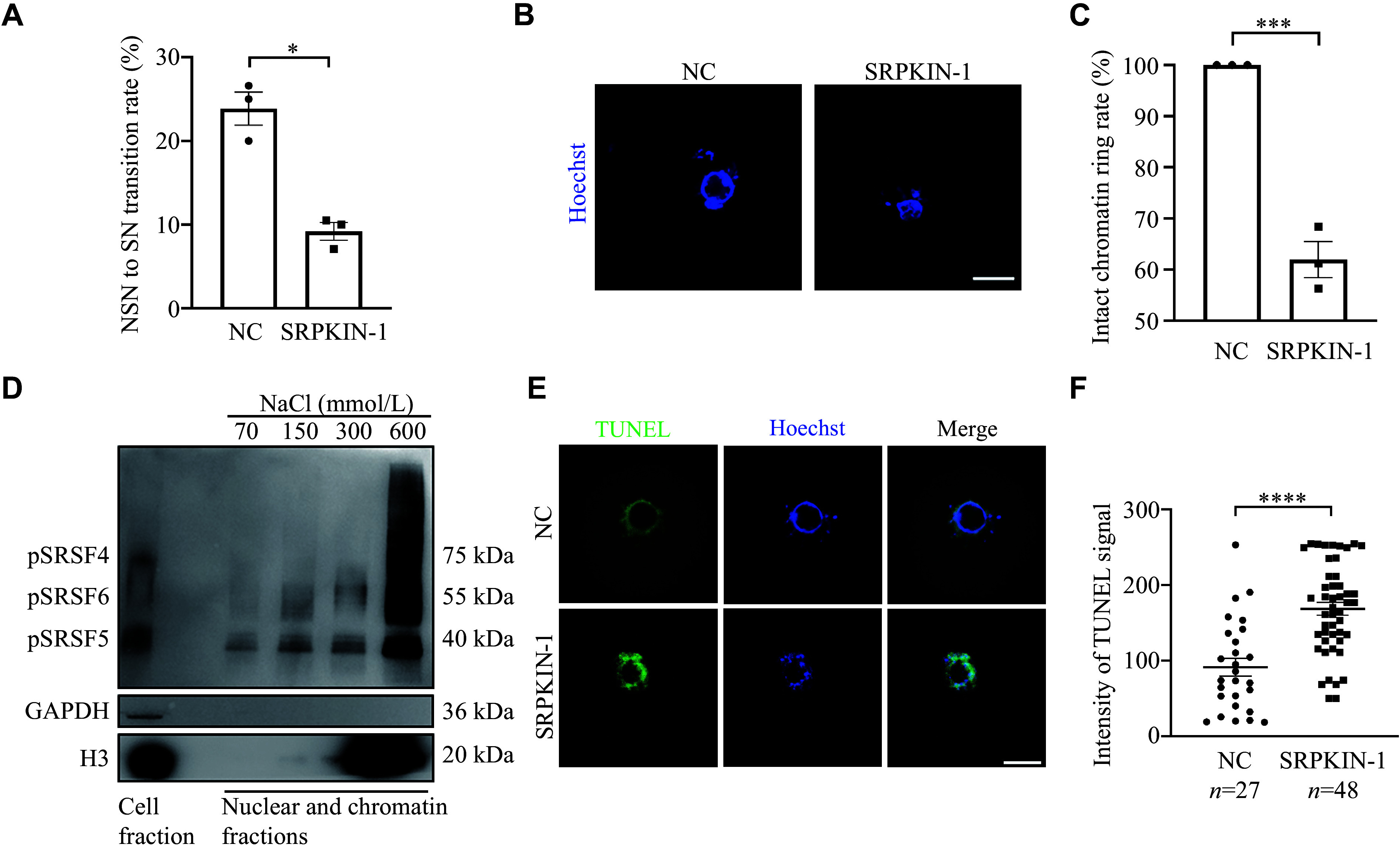
Phosphorylation of serine-arginine proteins was related to chromatin. A: Quantitative analysis of NSN to SN transition rate in NSN oocytes treated with SRPKIN-1 (50 μmol/L) or an equal volume of dimethyl sulfoxide (DMSO; NC) for 15 h. A total of 80 and 92 oocytes were counted in the NC and SRPKIN-1 treatment groups, respectively (*n* = 3 independent replicates). B: Representative images showing the chromatin state of SN oocytes treated with SRPKIN-1. Scale bar, 20 µm. C: Quantitative analysis on the rate of intact chromatin ring. A total of 80 and 77 SN oocytes were counted in the NC and SRPKIN-1 groups, respectively (*n* = 3 independent replicates). D: Western blotting analysis of pSR distribution on nuclear and chromatin fractions of Hek293 cells. GAPDH and H3 were used as cytosolic and chromatin markers, respectively. Numbers in nuclear fractions indicate the NaCl concentration at which the soluble fraction was extracted (*n* = 3 independent replicates). E: Representative images of TUNEL staining in SN oocytes with NC and SRPKIN-1 treatment. Scale bar, 20 µm. F: Quantification of TUNEL staining in NC and SRPKIN-1 treatment oocytes. Data are presented as mean ± standard error of the mean. ^*^P < 0.05, ^***^P < 0.001, and ^****^P < 0.0001, compared with the control group by unpaired two-tailed Student's *t*-test. Abbreviations: NSN, non-surrounding nucleolus; SN, surrounding nucleolus; SRPKIN-1, SR protein-specific kinase irreversible inhibitor 1; NC, negative control; H3, histone 3; TUNEL, terminal deoxynucleotidyl transferase-mediated dUTP-biotin nick end labeling assay.

Based on the above results, we hypothesized that inhibiting SRPK1 function in phosphorylating SR proteins would prevent the NSN-SN transition during oocyte development. To test this hypothesis, we cultured NSN oocytes *in vitro* in the medium containing SRPKIN-1. SRPKIN-1 treatment resulted in a significant reduction in the transition rate from NSN to SN oocytes, demonstrating the significance of pSR for oocyte development (***Fig. 4A***; ***Supplementary Fig. 2*** [available online]; and ***Supplementary Movie 1*** [available online]). Moreover, in SN oocytes cultured with SRPKIN-1 (***Supplementary Movie 2***, available online), it was observed that the perinucleolar rings collapsed, and the rate of defective rings increased significantly (***Fig. 4B*** and ***4C***, and ***Supplementary Fig. 2***). These findings led us to investigate the interaction between pSR and chromatin using the high salt solutions that can extract chromatin-bound proteins with histones from chromatin^[23]^. The results showed that pSR was eluted out with histone H3, particularly peaking at 600 mmol/L NaCl concentrations (***Fig. 4D***). Collectively, the above results suggest that pSR may bind to chromatin and participate in the NSN-SN transition.

### The phosphorylation of SR proteins was involved in nuclear condensation during the transition from NSN to SN oocytes

**Figure 5:**
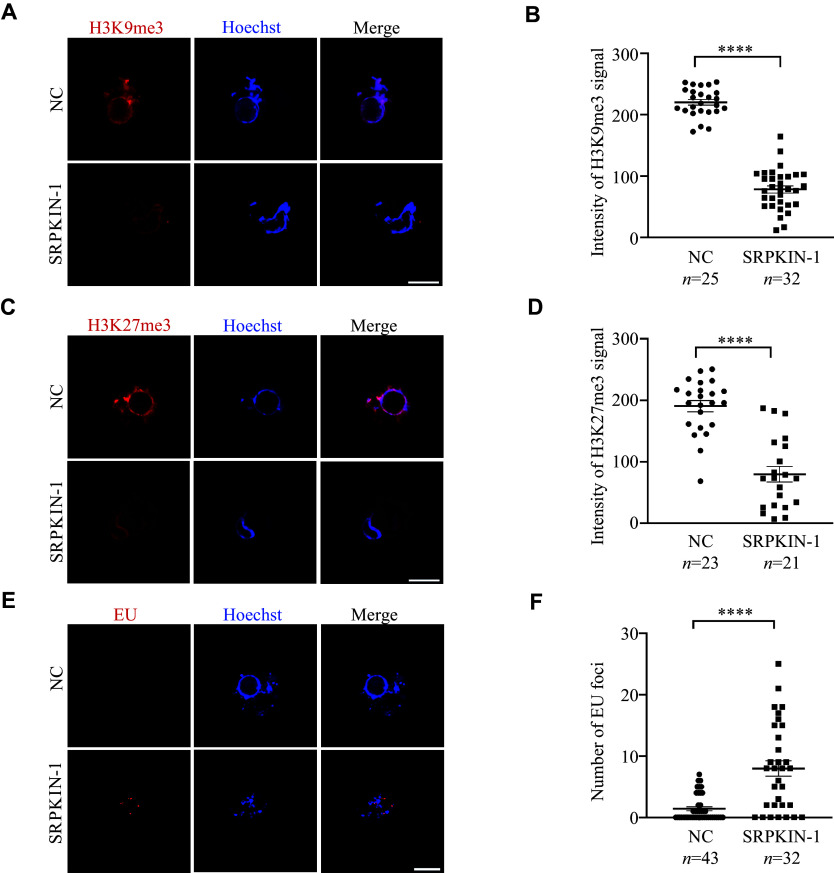
Phosphorylated serine-arginine proteins were involved in nuclear condensation. A–D: Representative images and quantification of H3K9me3 (A and B) and H3K27me3 (C and D) in SN oocytes treated with SRPKIN-1 (50 μmol/L) and an equal volume of dimethyl sulfoxide (DMSO; NC). E and F: Representative images and quantification of EU staining in SN oocytes treated with SRPKIN-1 (50 μmol/L) and an equal volume of DMSO (NC). Data are presented as mean ± standard error of the mean. ^****^P < 0.0001 in the unpaired two-tailed Student's *t*-test. All scale bars, 20 µm. Abbreviations: H3K9me3, histone H3 lysine 9 trimethylation; H3K27me3, histone H3 lysine 27 trimethylation; SRPKIN-1, SR protein-specific kinase irreversible inhibitor 1; EU, ethynyl uridine.

Considering the phenomenon of collapsed chromatin rings observed in SN oocytes cultured with SRPKIN-1, we speculated that the condensed chromatin became loose. To test this hypothesis, we performed an *in situ* DNase Ⅰ assay on SRPKIN-1 treated SN oocytes, where samples were incubated with DNase Ⅰ, followed by TUNEL assay. We found that the accessibility for the collapsed rings increased after the treatment of SRPKIN-1 in SN oocytes (***Fig. 4E*** and ***4F***), and no significant differences were observed in signal levels between the experimental and control groups without DNase Ⅰ treatment (***Supplementary Fig. 3A*** and ***3B***, available online). To be clear, γH2AX staining indicated that SRPKIN-1 treatment did not cause DNA damage (***Supplementary Fig. 3C*** and ***3D***). In addition, we analyzed the histone modifications of H3K9me3 and H3K27me3 by immunofluorescence staining (***Fig. 5A*** and ***5C***). Quantitative analysis showed that the fluorescence levels of these two modifications were both decreased, when SN oocytes were cultured with SRPKIN-1 (***Fig. 5B*** and ***5D***). The incorporation of the uridine analog ethynyl uridine showed a transcriptional signal appearing in the loosened chromatin (***Fig. 5E*** and ***5F***). Taken together, these results indicate that pSR plays an important role in the nuclear condensation during the transition from NSN to SN oocytes through its interaction with chromatin.

## Discussion

**Figure 6:**
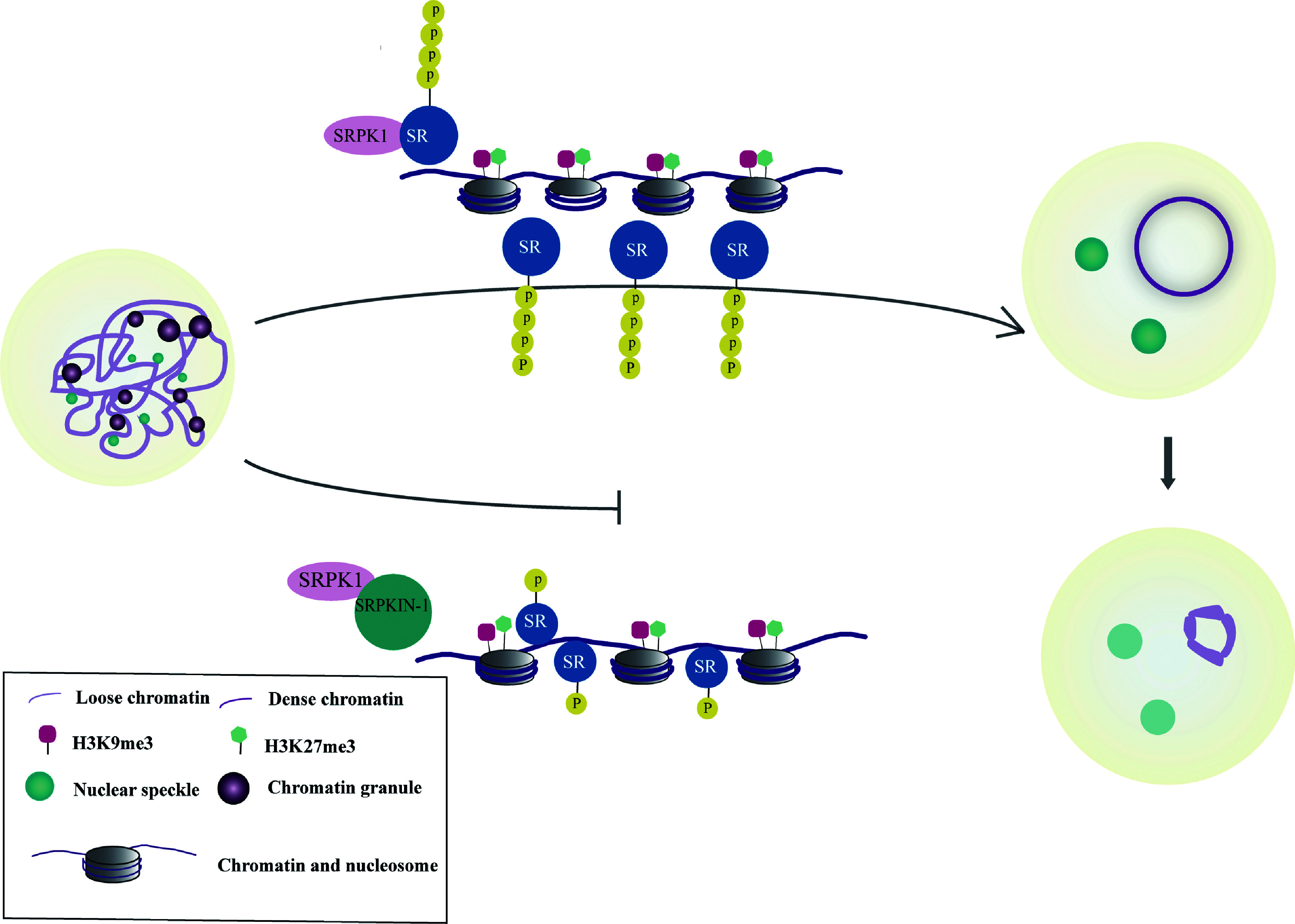
The relationship of serine-arginine (SR) proteins and chromatin in mouse oocytes. A schematic illustration showing that SR protein-specific kinase 1 (SRPK1)-mediated phosphorylation of SR proteins regulates the oocyte maturation through participating in the process of chromatin configuration change during non-surrounding nucleolus to surrounding nucleolus transition. Abbreviations: H3K9me3, histone H3 lysine 9 trimethylation; H3K27me3, histone H3 lysine 27 trimethylation.

In the current study, we demonstrated that NS spread over the nucleus in NSN oocytes and scattered as bright dots around the condensed chromatin in SN oocytes. The phosphorylation of SR proteins by SRPK1 was critical for oocyte development, through which the irreversible competitive inhibitor SRPKIN-1 blocked the transition from the NSN to the SN stage. Furthermore, when SN oocytes were treated with SRPKIN-1, the heterochromatin ring-shaped structure surrounding the nucleolus was demolished, along with more accessible chromatin, attenuated repressive histone modifications, and a re-emerging transcriptional signal (***Fig. 6***).

The traits of NS in NSN and SN oocytes are not clear. Al Jord *et al*^[24]^ suggested that the aggregation of NS from NSN to SN stage was caused by a gradual increase in cytoplasmic forces. Apart from the physiological cytoplasmic forces, NS also aggregates under some pathological conditions, such as protease inhibitors^[25]^ or transcription inhibitors^[26]^. We also found that the size of NS was altered and became more clustered when NSN oocytes were treated with SRPKIN-1. However, the role of NS clustering is still unclear, which is usually interpreted as being related to the splicing process^[24]^. By investigating the relationship between SR proteins and chromatin, the current study provides new insights into the function of NS aggregation in mouse oocytes.

As the first SR-specific kinase discovered, SRPK1 is highly conserved in eukaryotic cells^[27]^. We observed colocalization of NS and SRPK1 in the nucleus of SN oocytes, indicating the phosphorylation of SR proteins by SRPK1. However, the phosphorylation of SR proteins was significantly decreased in the SN stage, compared with the NSN stage. Studies have shown that hypo- or hyper-phosphorylated SR proteins are inactive in regulating RNA splicing^[28]^. The regulation of SR protein phosphorylation is required to control pre-mRNA splicing^[29]^, as reflected by its participation in spliceosome assembly^[30]^. Given the dramatic transcriptional fluctuation from NSN to SN oocytes^[5,7]^, it is reasonable to assume alterations in nascent RNA splicing, one of which is reflected by changes in phosphorylation of SR proteins, although the splicing at the oocyte developmental stage has not been precisely refined. In addition, cyclin-dependent-like-kinases (CLKs) colocalize with SR proteins in the nucleus^[31]^ and cooperate with SRPKs to catalyze and regulate SR proteins^[17]^, implying the phosphorylation of SR proteins by CLKs in NSN oocytes. The relevant conjectures need further verification.

In the current study, the levels of pSR significantly decreased when SRPKIN-1 was applied to cultured oocytes, resulting in a significant reduction in their developmental potential. Moreover, it was interesting to observe that the classic chromatin rings of SN oocytes were deconstructed when cultured with SRPKIN-1. A further sequential salt elution experiment proved the link between SR proteins and chromatin. Although NSs are generally considered interchromatin granules involved in transcriptional and post-transcriptional regulation, the correlation between NSs and chromatin has been gradually uncovered recently. NSs were shown to link to transcriptionally active loci through fluorescence *in situ* hybridization experiments^[32]^. It has been found that type A compartments, corresponding to transcriptionally active loci, tend to be close to NS by utilizing Hi-C for conformational analysis of chromatin interaction^[33]^. In the current study, chromatin accessibility increased, and transcriptional signals reappeared after a decrease of SR protein phosphorylation in SN oocytes, which disrupted the process of meiosis and decreased the quality of oocytes. This is because the complete cessation of transcriptional activity in SN oocytes is significantly correlated with the highly condensed chromatin state^[34]^.

The correlation between NS and chromatin indicates the regulation of downstream transcription. Raina *et al*^[35]^ demonstrated the stable relationship between NS and chromatin in HEK293 cells by disrupting the structure of chromatin, which brought about morphological changes in NS, but they did not further investigate whether the change in NS would, in turn, affect chromatin. In the current study, NS maintained its relationship with chromatin structure in SN oocytes, providing a new perspective for exploring intrinsic mechanism of the chromatin structure configuration transition during oocyte maturation.

It should be noted that the molecular mechanisms underlying the interaction between pSR and oocyte chromatin remain unexplored. In addition, the role of phosphorylation of SR proteins in physiological oocytes remains unknown, because it manifests as a block phenotype under the influence of the inhibitor. These questions need future investigation. In summary, the current study demonstrated that NS was substantially agglomerated during the NSN-SN transition. When the phosphorylation of SR proteins was hindered by the SRPK1 inhibitor SRPKIN-1, oocyte meiotic maturation was disrupted, the transition from NSN to SN was reduced, and the chromatin rings in SN oocytes collapsed. Mechanistically, we further found that pSR displayed a strong bond with chromatin and contributed to the oocyte maturation process by maintaining the classic chromatin structure.

## SUPPLEMENTARY DATA

Supplementary data to this article can be found online.
