# Supplementary material for: The role of SRPK1-mediated phosphorylation of SR proteins in the chromatin configuration transition of mouse germinal vesicle oocytes
Source: J Biomed Res. 2024 May 29;39(2):198–208. doi: 10.7555/JBR.38.20240054 (PMC11982682; doi:10.7555/JBR.38.20240054)
Supplement: Supplementary file 1 — Supplementary data to this article can be found online. [file jbr-39-2-198-S1.pdf]

Available online at www.jbr-pub.org.cn
Open Access at PubMed Central
Journal of Biomedical Research, 2025 39(2): 1–3
Supplementary Data
JBR

The role of SRPK1-mediated phosphorylation of SR proteins in the chromatin configuration transition of mouse germinal vesicle oocytes

Xia Wang[1,△], Shuai Zhou[2,△], Haojie Yin[1], Jian Han[1], Yue Hu[1], Siqi Wang[1], Congjing Wang[1], Jie Huang[1], Junqiang Zhang[2], Xiufeng Ling[2,✉], Ran Huo[1,✉]

[1]*State Key Laboratory of Reproductive Medicine and Offspring Health, Department of Histology and Embryology, Suzhou Affiliated Hospital of Nanjing Medical University, Suzhou Municipal Hospital, Gusu School, Nanjing Medical University, Nanjing, Jiangsu 211166, China;*
[2]*Department of Reproductive Medicine, Women's Hospital of Nanjing Medical University, Nanjing Women and Children's Healthcare Hospital, Nanjing, Jiangsu 210004, China.*

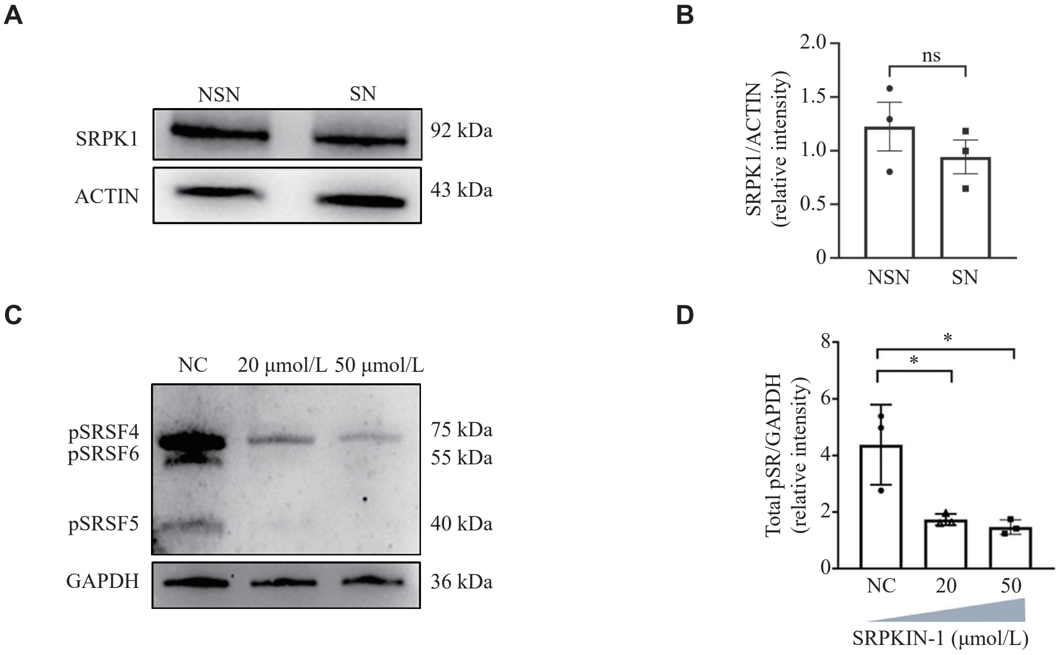

(Continued)

[△]These authors contributed equally to this work.

[✉]Corresponding authors: Ran Huo, State Key Laboratory of Reproductive Medicine and Offspring Health, Department of Histology and Embryology, Suzhou Affiliated Hospital of Nanjing Medical University, Suzhou Municipal Hospital, Gusu School, Nanjing Medical University, 101 Longmian Road, Nanjing, Jiangsu 211166, China. E-mail: huoran@njmu.edu.cn; Xiufeng Ling, Department of Reproductive Medicine, Women's Hospital of Nanjing Medical University, Nanjing Women and Children's Healthcare Hospital, 123 Tianfei Lane, Nanjing, Jiangsu 210004, China. E-mail: lingxiufeng_njfy@163.com.

Received: 01 March 2024; Revised: 06 May 2024; Accepted: 10 May 2024; Published online: 29 May 2024

CLC number: R321.1, Document code: A

The authors reported no conflict of interests.

This is an open access article under the Creative Commons Attribution (CC BY 4.0) license, which permits others to distribute, remix, adapt and build upon this work, for commercial use, provided the original work is properly cited.

© 2025 by Journal of Biomedical Research.

https://doi.org/10.7555/JBR.38.20240054

(Continued)

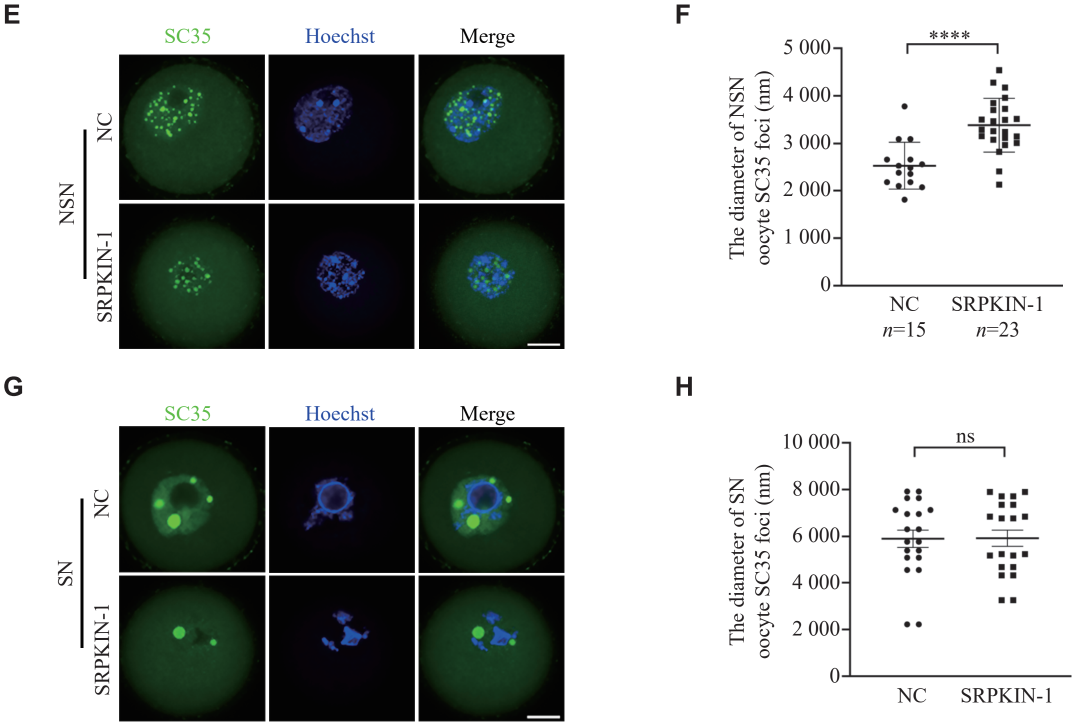

Supplementary Fig. 1 **Changes of NS in oocytes after SRPKIN-1 treatment.** A: Immunoblot of SRPK1 expression in denuded freshly NSN and SN oocytes. ACTIN served as the loading control. B: Quantification of the grayscale from panel A (n = 3 independent replicates). C: Western blotting showing the distribution of pSR with SRPKIN-1 at concentrations of 20 μmol/L and 50 μmol/L. GAPDH served as the loading control. D: Quantification of the grayscale from panel C (n = 3 independent replicates). E: Representative images of SC35 staining showing the morphology of NS after inhibitor treatment in NSN oocytes. F: Quantification of the diameter of SC35 signal in NSN oocytes. G: Representative images of SC35 signal in SN oocytes with inhibitor treatment. H: Quantification of the diameter of SC35 signal in SN oocytes. Data are presented as mean ± standard deviation of the mean. ns, not significant. $^{*}P < 0.05$ and $^{****}P < 0.000\ 1$ by unpaired two-tailed Student's t-test. All scale bars, 20 μm. Abbreviations: NS, nuclear speckle; SRPK1, SR protein-specific kinase 1; NSN, non-surrounding nucleolus; SN, surrounding nucleolus; pSR, phosphorylated SR; SRPKIN-1, SR protein-specific kinase irreversible inhibitor 1.

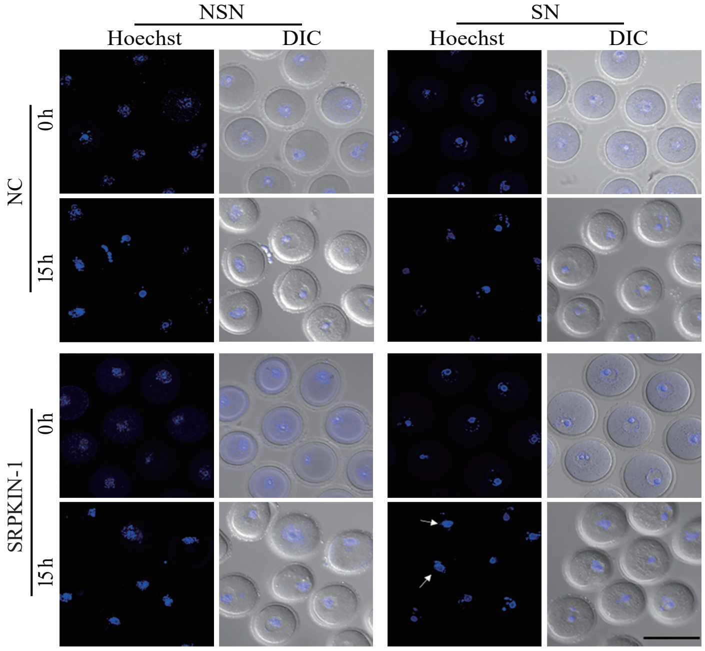

Supplementary Fig. 2 **Changes in chromosomal status in oocytes after SRPKIN-1 treatment.** Representative Hoechst staining and differential interference contrast (DIC) images showing the development of non-surrounding nucleolus (NSN) and surrounding nucleolus (SN) oocytes at 0 h and 15 h of *in vitro* culture with serine-arginine protein-specific kinase irreversible inhibitor 1 (SRPKIN-1; 50 μmol/L). Arrowheads represent the abnormal chromatin rings in the treatment group. Scale bar, 100 μm.

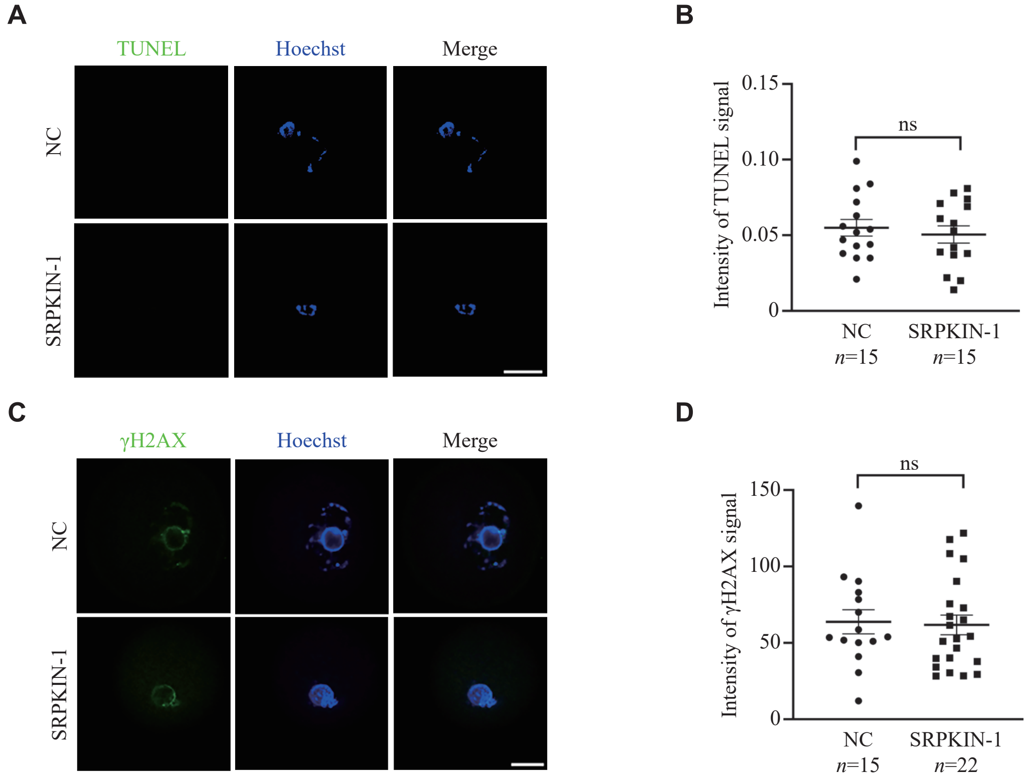

Supplementary Fig. 3 **Detection of oocyte physiological status after the SRPKIN-1 treatment.** A: Representative images of TUNEL staining in the NC and SRPKIN-1 treatment groups. B: Quantification of TUNEL intensity in NC and SRPKIN-1 treatment oocytes. C: Representative images of γH2AX staining showing the level in control and SRPKIN-1 treatment oocytes. D: Quantification of γH2AX staining in NC and SRPKIN-1 treatment oocytes. Data are presented as mean ± standard deviation of the mean. ns, not significant. All scale bars, 20 μm.

Supplementary Movie 1

Live-cell imaging of differential interference contrast (DIC) and Hoechst staining showing the chromatin state in non-surrounding nucleolus (NSN) oocytes between the negative control (NC) and SRPKIN-1 treatment groups. Time (h) represents the time of *in vitro* culture. Scale bar, 20 μm.

Supplementary Movie 2

Live-cell imaging of differential interference contrast (DIC) and Hoechst staining showing the chromatin state in surrounding nucleolus (SN) oocytes between the negative control (NC) and SRPKIN-1 treatment groups. Time (h) represents the time of *in vitro* culture. Scale bar, 20 μm.
